# The Role of the Simulation in Supporting Newly Graduated Nurses in Their First 5 Months of Working Transition: Findings From a Mixed‐Method Study

**DOI:** 10.1111/jocn.17617

**Published:** 2024-12-12

**Authors:** Maura Mesaglio, Sara Dentice, Luca Grassetti, Illarj Achil, Anna Bernardinis, Davide Caruzzo, Anna Inserra, Irene Mansutti, Elisa Mattiussi, Sandra Menegoz, Tommaso Piani, Elena Vanzo, Stefania Chiappinotto, Alvisa Palese

**Affiliations:** ^1^ Azienda Sanitaria Universitaria Friuli Centrale Udine Italy; ^2^ Department of Medicine University of Udine Udine Italy; ^3^ Italian National Institute of Health Rome Italy; ^4^ Department of Economics and Statistics University of Udine Udine Italy; ^5^ Azienda Sanitaria Friuli Occidentale Pordenone Italy

**Keywords:** newly graduated nurses, nursing practice, preceptors, professional development, self‐assessed competencies, simulation, work transition

## Abstract

**Aims:**

To understand the role of simulation in ensuring the development of the competencies expected by newly graduated register nurses (NGRNs) from the work initiation up to 5 months of transition.

**Methods:**

Mixed‐method study design. A longitudinal phase employing the Nurse Competence Scale (NCS, from 0 to 100, excellent) to assess the perceived competencies among NGRNs (*N* = 151) at three time points (first day of work up to fifth month); followed by a qualitative phase involving four focus groups of preceptors (*N* = 16) to explore the potential role of simulation in the NGRNs' working transition. Integration was performed at findings level, using the building procedures and joint displaying the results.

**Results:**

During the different time periods, variations emerged in the NCS scores from 64.41 out of 100 in the first day of work to 61.82 after 15 days, reaching 69.25 and 73.21 at 3 and 5 months. Nine potentialities have been identified as having simulation supporting NGRNs during their transition to independent practice. Simulation may contribute to develop competencies in some competence domains (diagnostic function, managing situation, therapeutic intervention, quality assurance and working role) while not in others (helping role and teaching–coaching).

**Conclusion:**

Early interventions, through integration of simulation sessions into strategies offered at the unit's level may be useful to ensure an effective working transition.

**Impact:**

*Problem the study addresses*: Challenges in transition from education to working settings are increasing given the difficulties of the units in providing time and support to NGRNs. *Main findings*: Competencies of NGRNs' are fluctuant in the five first months of work, and sub‐optimal in certain domains. Simulation may support the full development of most competencies. *Impact on research*: Healthcare organisations can support NGRNs to ensure smoother transitions by integrating simulations in their strategy.

**Reporting Method:**

This study was conducted following the Good Reporting of a Mixed‐Methods Study.

**Patient or Public Contribution:**

Only healthcare professionals were involved.

**Summary**

- What does this paper contribute to the wider global clinical community?
  ○ Longitudinal analyses show fluctuations in new graduate registered nurses perceptions of competence in their transition to independent practice, suggesting the need of tailored support.
  ○ Simulation is identified as an important resource for competence development of NGRNs, providing learning opportunities in a safe environment and helping to bridge the gap between education and clinical practice.
  ○ Simulation accessibility, with diverse intensity, from high to lower, delivered in the first months of the working experience, as integrated within the unit were the main strategies suggested.

## 1 Introduction

The transition from academia to independent practice is a critical time for newly graduated registered nurses (NGRNs). This transition has been defined as a ‘*nonlinear experience*’, causing NGRNs to face personal and professional, intellectual and emotive and skill and role relationship changes where experiences, meanings and expectations play an important role (Duchscher 2008). While education should establish a robust theoretical nursing foundation, it is within the work environment that NGRNs are confronted with complex situations where they are expected to demonstrate practical skills and adaptation to the real dynamics of the workplace (Aldosari, Pryjmachuk, and Cooke 2021). During this phase, NGRNs are confronted with the so‐called reality shock, produced by the differences between the theoretical knowledge acquired during education and the complex, often unpredictable demands of real‐world clinical practice (Labrague 2024). Moreover, they are also pressured by the expectations of experienced nurses to quickly acquire practical skills and integrate them into daily practice (Kaihlanen, Lakanmaa, and Salminen 2013). As a result, NGRNs may feel unprepared and suffer from higher levels of anxiety and stress, making this transition one of the most challenging phases in a nurse's career (Holtz et al. 2024). The quality of the work environment, the patients' and family expectations in addition to that of experienced nurses and healthcare team may influence the psychological well‐being of NGRNs, moderating their perceived stress (Reebals, Wood, and Markaki 2021). When not well addressed, the transition may have a significant impact on professional and personal development and even lead to the decision to leave the profession (Edwards et al. 2015), documented in approximately 25% of new graduates (Schmitt and Schiffman 2019).

The NGRNs transition process has been divided into different stages, in which after five‐months there is a significant shift in the professional growth, corresponding to the attainment of consolidation and the construction of meaning (Duchscher 2008). Therefore, the first months play a crucial role in allowing NGRNs to learn from more experienced colleagues, as well as from specific training programmes and from dealing with a variety of clinical cases. However, the shortages of nurses prevent the on‐the job training, preceptorship and supervision, forcing NGRNs to demonstrate an independent practice immediately (Bassi et al. 2023). As a consequence, a clear strategy to support nurses during this transition has been recommended to improve patient's care quality and to foster professional growth and retention (See et al. 2023). In this context, simulation‐based learning can bridge the gap between theoretical knowledge and clinical practice and improve self‐reported confidence, competence and readiness for practice (Opoku, Khuabi, and Van Niekerk 2021). However, the evidence available have been obtained from small samples, using unvalidated tools, and mainly with cross sectional studies recommending further investments in this field (Harper, Bodine, and Monachino 2021). Therefore, the intent of this study was to expand the evidence available in the context of simulation and NGRNs working transition.

## 2 Background

The NGRNs competencies are defined as the set of knowledge, skills and attitudes that enable them to perform tasks effectively in the clinical environment and deliver quality patient care. Specifically, (a) knowledge includes a strong grasp of both theoretical and practical aspects (e.g., anatomy and pharmacology), as well as those regarding holistic care, in order to address not only physical, but also psychological and cultural needs of patients; (b) skills involve technical tasks (e.g., administering medications, monitoring vital signs) and critical thinking to make effective decisions; moreover, interpersonal abilities, such as communication and teamwork, are also considered essential to ensure collaboration and patient interaction; and (c) attitudes emphasise professionalism, empathy and a commitment to continuous learning. NGRNs must approach their roles with compassion and a dedication to ethical care, fostering patient trust and ensuring high‐quality outcomes. The possession of all competencies improves the capacity of NGRNs to face the challenges of the clinical environment (Rabie, Rabie, and Dinkelmann 2020).

The evaluations of such competencies during the NGRNs transition from working initiation up to 5 months (Duchscher 2008) are important to identify educational needs (Byermoen et al. 2023). Such evaluations may be based on observed performance as detected by experienced nurses/preceptors (Taylor et al. 2021) or through self‐assessment, enabling NGRNs to reflect on competencies, strengths, weaknesses and areas for improvements as well as on their professional identity and role within the healthcare team (Clemett and Raleigh 2021). Predominantly used during the work transition, self‐assessment contributes to tailoring educational strategies to develop the required competencies (Numminen et al. 2016); moreover, when used longitudinally, it may further track competence evolution over time, providing information about the effectiveness of the educational strategies adopted (Lima et al. 2016; Lindfors et al. 2022). However, studies available have mainly used NGRNs self‐assessments concerning the start of their work (e.g., Wangensteen et al. 2012) and few studies have extended the data collection over time (e.g., Lima et al. 2016). Moreover, self‐assessment may result in under or overestimation of the competencies possessed (Huang 2013): thus, data provided by external observers, such as nurse preceptors, may help accurately identify the educational needs. However, only in few studies (Prion et al. 2015) the preceptor evaluations of the observed performance have been integrated into the self‐assessment measures collected from NGRNs.

According to the level of competence measured, in order to protect patients from unsafe practices, the healthcare system should offer tailored strategies that may range from on‐site educational sessions designed to develop on‐the‐job competencies to clinical simulations provided at simulation centres, delivering dynamic and engaging learning experiences. Using high‐fidelity mannequins, advanced software and immersive simulations, NGRNs can deal with complex scenarios and realistic clinical situations without compromising patient safety (Yeh et al. 2022; Yuan et al. 2012). Simulation enables NGRNs to gain progressive confidence, to cope with stressful situations and to develop the skills needed to handle emergencies or complex procedures, as well as to deal with situations that may not occur frequently (Sterner et al. 2022). Moreover, the interactivity of simulation sessions allows to receive immediate and personalised feedback that may improve the performance. Therefore, simulation sessions may be considered crucial not only among undergraduate students but also in the transition process of NGRNs (Sim et al. 2022). However, no data are available regarding the potential role of simulation in supporting, refining or advancing the self‐perceived competencies among NGRNs over time by integrating the perspectives of new graduates and that of their preceptors.

## 3 The Study

To understand the role of simulation in ensuring the development of the competencies expected by NGRNs from the work initiation up to 5 months of transition.

## 4 Methods

### 4.1 Study Design

A mixed‐method study design composed by a longitudinal study and a nested descriptive qualitative study was conducted from November 2022 to June 2023 following the Good Reporting of a Mixed‐Methods Study (O'Cathain, Murphy, and Nicholl 2008) (Table S1). According to Creswell and Clark (2018) this mixed‐method study was based on the following characteristics.

1. Sequence:

- a quantitative method, based on a longitudinal study design was used to collect the perceived competencies as self‐assessed by NGRNs over the time; and
- a descriptive method, based on a descriptive qualitative study design involving focus groups was used to collect perceptions of preceptors regarding the potential role of the simulation in supporting NGRNs in the transition from their graduation up to the first 5 months.

2 Priority: the qualitative method prevailed in terms of weight throughout the research process (‘quan➔QUAL’) (Morse 1991);
3 Integration: data integration was performed combining findings collected in the first and second phase (Greene, Caracelli, and Graham 1989; Schoonenboom and Johnson 2017).

### 4.2 Setting and Sampling

Two teaching hospitals in North‐eastern Italy were approached and accepted to participate. They were equipped with 800 and 1000 beds respectively, located in the same region, and therefore subject to the same policies and rules for NGRNs recruitment, competence assessment system and job placement mechanisms. Given the lack of nursing resources, only a few days of supervision by preceptors were ensured during the transition phase from the first day of work up to the fifth month, and NGRNs were asked to become independent as soon as possible.

The following sampling methods were used.

- Quantitative method: All hired NGRNs initiating their work in the above‐mentioned teaching hospitals were eligible. Those NGRNs with no previous work experience, working in the same unit since their recruitment and willing to participate in the study, were all included. Consequently, those who had ceased to work in the unit or hospital were excluded. To ensure anonymity, the reasons for work cessation were neither asked nor explored. A total of 209 NGRNs were recruited; given that 58 reported previous working experiences, these were excluded. A total of 151 NGRNs were eligible and agreed to participate in the study.
- Qualitative method: A purposeful sample of nurses who had been certified as preceptors (Amaral and Figueiredo 2023) in medical, surgical, critical care and paediatric settings, and who were engaged in mentoring the included NGRNs, was identified. Specifically, 16 nurses accepted to participate (all those contacted): 6 from critical care, 4 from medical, 3 from surgical and 3 from paediatric settings. Most nurses were female (15; 93.7%) with an average age of 41 years.

### 4.3 Data Collection and Procedures

The following data sources were used.

- Quantitative method: A longitudinal data collection was performed by using the Nurse Competence Scale (NCS) (Meretoja, Isoaho, and Leino‐Kilpi 2004) according to its documented validity and reliability in measuring the self‐perceived competencies (Flinkman et al. 2017; Kajander‐Unkuri et al. 2021). In the present study, the internal consistence of the tool was (*α* Cronbach) T0 = 0.992; T1 = 0.992; T3 = 0.988; T4 = 0.983. The NCS included 73 items divided into seven dimensions: (a) helping roles (*n* = 7 items), (b) teaching and coaching (*n* = 16), (c) diagnostic functions (*n* = 7), (d) situation management (*n* = 8), (e) therapeutic interventions (*n* = 10), (f) quality assurance (*n* = 6) and (g) working roles (*n* = 19). The tool measures the perceived levels of competence using a visual analogue scale (VAS) ranging from 0 (low level) to 100 (high level of competence). For the purposes of this study, scores below 60 indicate ‘insufficient competence’, higher than 60 up to 75 ‘sufficient’ and over 75 ‘good competence’ (Table 1); some demographic data were also collected.
  Data were collected at T0 (first day of work as a NGRNs); at T1 (after 15 days), at T2 (after 3 months) and at T3 (after 5 months) according to the available literature (Duchscher 2008). For each NGRNs, an identification code was created to track the self‐assessments over time and ensure confidentiality.
  The data collection was conducted by utilising the European Commission platform (EUSurvey), known for its adherence to privacy and confidentiality (European Commission 2022). To ensure participation, the survey was sent on the scheduled day to each NGRNs. Two reminders were subsequently sent within a period of about one month after the expiry of T1, T2 or T3. To some of them, a telephone reminder was also provided.
- Qualitative method: four focus groups (one in each setting where the included NGRNs were hired, namely, medical, surgical, critical care and paediatric units) were conducted. These were performed over a period of 10 days, in one single session per each unit, at the end of the transition time, 5 months after the study initiation. The focus groups were conducted in a calm environment (hospital rooms, close to the unit); each was managed by experienced nurses with a research background (see authors), external to the hospital. All those invited accepted to participate and their informed consent was collected before the focus group initiation. Moreover, the focus group discussion was conducted by using mainly open‐ended questions (Table 1); the debate was moderated by the second researcher and audio recorded. In order to ensure a free discussion, NGNRs were not involved and researchers were not aware of which new graduates were supervised by each preceptor.

**TABLE 1:** Data collection tools in quantitative and qualitative studies: Main characteristics.

Quantitative phase. To evaluate the overall competencies as perceived by New Graduated Regiestered Nurses (NGRNs) to perform tasks and integrate knowledge, skills, attitudes, and values effectively in various specific situations, the Nurse Competence Scale (NCS) was used. The tool has been developed by Meretoja, Isoaho, and Leino‐Kilpi (2004) and validated in the Italian language (Dellai, Mortari, and Meretoja 2009; Finotto and Cantarelli 2009; Notarnicola et al. 2018). The tool includes 73 statements divided into seven domains: Helping role: involves establishing a therapeutic connection by offering solace, being actively present, enabling empowerment, communicating through physical contact, and guiding individuals through transformative experiences (seven items). Teaching–coaching: identifies the will to acquire knowledge, promote the assimilation of illness and healing in daily life, make people understand, and explain and orientate (16 items); Diagnostic functions: involves assessment, identification of altered conditions, prediction of potential problems, understanding of diseases, and recognition of recovery potential (seven items). Managing situations: identifying and reacting to a declining or worsening situation (eight items). Therapeutic interventions: actions implemented by nurses to oversee a patient's illness, condition, or injury and prevent additional clinical interventions (10 items). Ensuring quality: includes risk prevention, quality and safety assurance, engagement in critical thinking and reflection, and the integration of evidence‐based practice and research (six items); Work role: managing requirements, setting priorities, and collaborating efficiently within a team (19 items). A few demographic data (age, gender) were also collected with a form. Qualitative phase. Data were collected through focus groups (*n* = 4) with the following three main open‐ended questions *Introduction questions* 8 What competencies are possessed by NGRNs as perceived by you, in your unit, and according to your preceptor role as enacted in the last five months? 9 What competencies do you think require more support to be developed by NGRNs during their transition? and *Core questions* What is the potential role of simulation to fill in the gaps in the competencies possessed by NGRNs in the transition phase? What are the best strategies to implement the simulation during the NGRNs transition phase?

### 4.4 Data Analysis

The following procedures were used.

- The quantitative analyses were based on descriptive statistics. The conditional means and the confidence intervals (CI) at 95% over time and the averages compared with paired test were calculated. The patterns of the seven dimensions as measured with the NCS were also conveyed considering the box plot representation. All the analyses have been developed in R using basic functions and the library ggplot2 for the graphical representations and the findings were considered statistically significant when *p* < 0.05. Particular attention was paid to the graphical representation of observations to give a clear view of time patterns for the dimensions and of sample size drop over the observational period. To this end, the box plot representation of scales in the four time periods was superposed with the jittered distribution of the observations to give a direct vision of sample distribution.
- The qualitative analysis of the data collected through the focus groups was based on the thematic analysis of the narratives verbatim transcribed (Braun and Clarke 2006). The narratives have been familiarised, examined and then categorised by two researchers (SD and AP) according to the core question (Table 1) and agreed upon by the research group (see authors). Quotes have been extracted in order to provide essential support in the categories identified, by also providing an identification code (e.g., MA, Focus group of the Medical Area). The process was conducted manually by two researchers and disagreements were discussed with a third researcher (SC). The trial code is provided in the Table S2.

Subsequently, the integration phase (Greene, Caracelli, and Graham 1989; Schoonenboom and Johnson 2017) was performed by following the building technique (Younas, Pedersen, and Durante 2020): the themes emerged in the focus groups were classified into the domains of the NCS according to their contents (Meretoja, Isoaho, and Leino‐Kilpi 2004), in order to identify (a) the competencies susceptible of simulation and (b) the degree of fit between the data collected in quantitative and qualitative phase (Greene, Caracelli, and Graham 1989; Schoonenboom and Johnson 2017). Thus, the joint displayed created combining the themes emerged and the domains of the NCS competencies were completed with the self‐assessments provided over time by NGRNs resulting in insufficient, sufficient and good competencies. The visual representation allowed to identify the appropriate competence that may benefit from simulation in the different stages of transition. The entire process was conducted by two researchers (SD and AP) and then checked by a third researcher (SC). The legitimation criteria are reported in Table S3.

### 4.5 Ethical Considerations

The research project was approved by the Institutional Review Board of the Department of Medicine, University of Udine, Italy (154/2022).

- Quantitative method: Since the first working months are considered by the law as those under evaluation, that is, if nurses do not show sufficient competencies, they are removed, personal data were not collected to ensure protection. Participation was on a voluntary basis and the written consent was collected by a contact person in each hospital; participants were allowed to withdraw from the study at any time without any consequence; moreover, no rewards were offered to those who participated. After the informed consent was signed, participants received the online questionnaire, sent to the email addresses given by the hospital. They were allowed to fill in the questionnaires during their working hours.
- Qualitative method: Those nurses appointed to be preceptors of the included NGRNs were contacted and explained the aims and the procedures of the study. They were free to either participate or withdraw from the study at any time without any consequence. After their acceptance to the study participation, data collection was performed during their working time.

## 5 Results

### 5.1 Quantitative Method: Self‐Assessment Competencies Over Time

There were involved 151 NGRNs with an average age of 23.6 (95% CI 23.3–24.9) and mainly female (119; 78.8%). Around half of them (73; 48.3%) responded at T1 and one‐third (51; 33.7%) at T2; and 32 of them (21.2%) participated up to the end of the follow‐up (Table 2).

**TABLE 2:** Flow diagram of participants.

	T0	T1	T2	T3
Participants	151	73	51	32
Dropouts from T0 (%)		78 (51.7)	100 (66.2)	119 (78.8)
Dropouts from the previous T (%)			22 (30.1)	19 (37.3)

*Note:* T0: day of starting work. T1: after 15 days. T2: after 3 months. T3: after 5 months.

During the different time periods (T0, T1, T2 and T3), variations emerged in the NCS scores, showing as compared to the T0 (64.41 out of 100; 95% CI 61.4, 67.43), an initial decrease at T1 in all dimensions (61.82; 95% CI 57.85–65.80) and an increase at T2 (69.25; 95% CI 64.87–73.64) and at T3 (73.21; 95% CI 67.82–78.61) (Table 3). ‘Helping role’ was the competence ranked by NGRNs at the highest scores over time, starting at 68.61 out of 100 (95% CI 65.70–71.52) at T0 and reaching 77.41 (95% CI 71.58–83.25) at T3. In contrast, ‘therapeutic interventions’ was the competence reporting the lowest scores, starting from 59.15 out of 100 (95% CI 55.76–62.53) at T0 and reaching 69.97 (95% CI 63.89–76.05) at T3 (Table 3). Statistical differences in the scores across comparisons over time have emerged, suggesting that, in nearly all cases, the self‐assessed competencies had significantly improved after 3 months, reaching higher values at 5 months (Table 3).

**TABLE 3:** Newly graduates registered nurses’ self‐assessment with the Nurse Competence Scale over time.

Dimensions, scores,^a^ mean (CI 95%)	T0	T1	T2	T3	T0 vs. T1 (*df* 66)	T0 vs. T2 (*df* 47)	T0 vs. T3 (*df* 31)	T1 vs. T2 (*df* 35)	T1 vs. T3 (*df* 21)	T2 vs. T3 (*df* 24)
	*N* = 151	*N* = 73	*N* = 51	*N* = 32						
1. Helping role	68.61 (65.7; 71.52)	68.12 (64.55; 71.69)	76.16 (72.8; 79.53)	77.41 (71.58; 83.25)	0.720	0.031	0.199	< 0.001	0.139	0.004
2. Teaching–coaching	63.2 (60; 66.4)	60.08 (55.84; 64.31)	65.76 (60.99; 70.53)	68.30 (60.88; 75.72)	0.132	0.515	0.645	0.001	0.246	0.004
3. Diagnostic functions	64.6 (61.36; 67.83)	62.64 (58.44; 66.84)	70.64 (65.89; 75.4)	75.13 (69.21; 81.06)	0.162	0.062	0.016	< 0.001	0.001	0.002
4. Managing situations	64.54 (61.37; 67.72)	61.82 (57.6; 66.03)	69.58 (64.79; 74.38)	73.83 (67.68; 79.98)	0.042	0.034	0.011	< 0.001	< 0.001	0.023
5. Therapeutic interventions	59.15 (55.76; 62.53)	56.12 (51.32; 60.93)	64.47 (59.29; 69.65)	69.97 (63.89; 76.05)	0.077	0.136	0.022	< 0.001	< 0.001	0.004
6. Ensuring quality	65.68 (62.31; 69.06)	62.1 (57.66; 66.54)	70.07 (64.88; 75.25)	74.27 (67.63; 80.91)	0.103	0.199	0.241	< 0.001	0.003	0.083
7. Working role	65.11 (62.06; 68.16)	61.87 (58.02; 65.73)	68.08 (63.41; 72.75)	73.59 (68.94; 78.23)	0.022	0.270	0.022	< 0.001	< 0.001	0.001
Overall	64.41 (61.40; 67.43)	61.82 (57.85; 65.8)	69.25 (64.87; 73.64)	73.21 (67.82; 78.61)	0.080	0.102	0.061	< 0.001	0.001	< 0.001

*Note:* T0: first day of work. T1: after 15 days. T2: after 3 months. T3: after 5 months; T0 vs. T1, T0 vs. T2, T0 vs. T3, T1 vs. T2, T2 vs. T3 comparison for paired data of scores over time, *p*‐value.

Abbreviations: CI, confidence interval; *df*, degrees of freedom; *N*, number.

^a^ The Nurse Competence Scale, at the competence level, is measured on a visual analogue scale, where 0 indicates a very low level and 100 indicates a high level of competency. For the purposes of this study, scores below 60 indicate “insufficient competence”, > 60 up to 75 “sufficient” and over 75 “good competence”.

**FIGURE 1:**
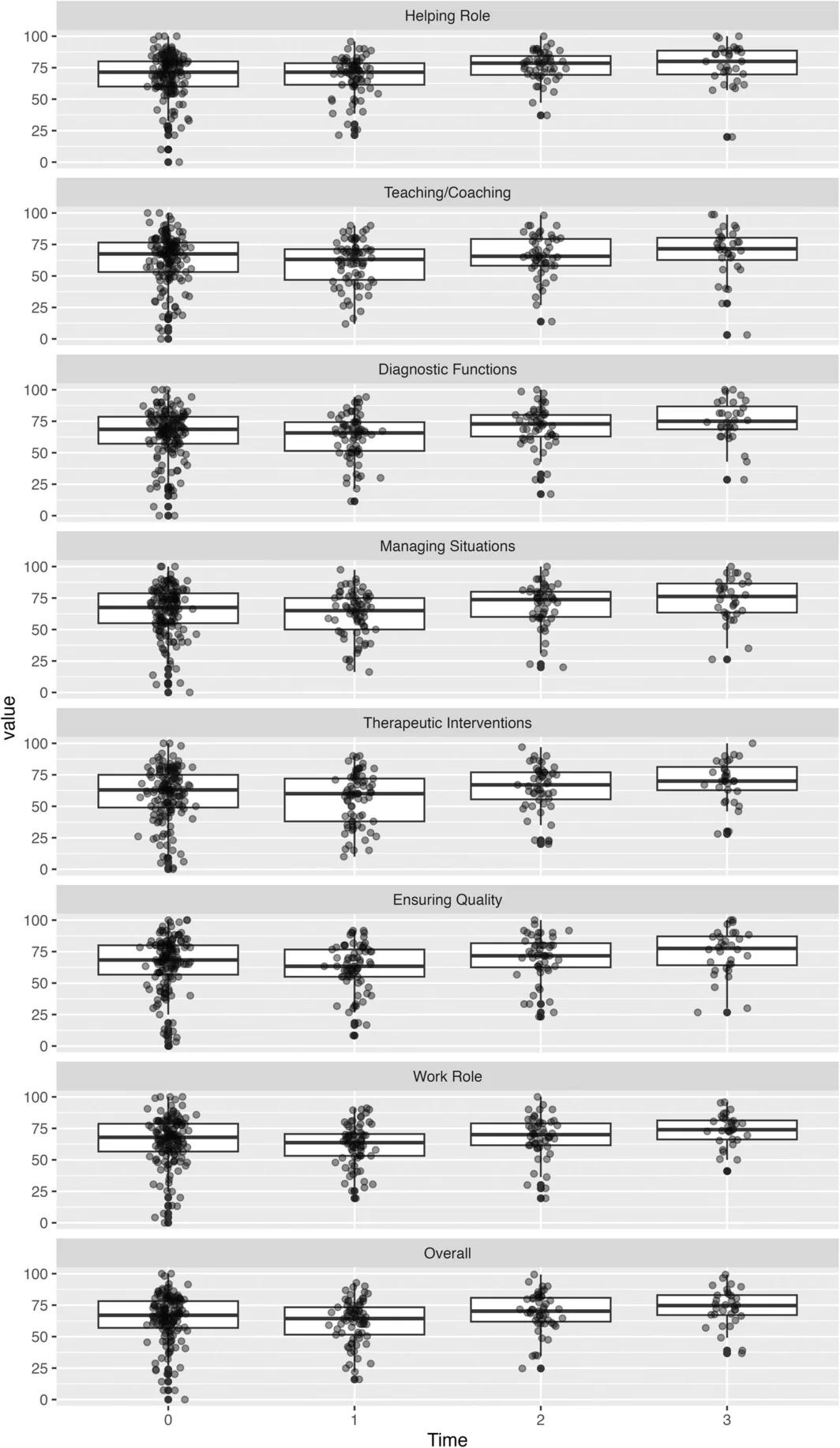
Boxplot NGRNs NCS self‐assessments. NCS, Nursing Competence Scale; NGRNs, New Graduated Registered Nurses.

From the analysis of the data distribution, at T0 and T1, the self‐assessment scores of all competencies have reported a wider distribution than at T2 and T3, when these were more concentrated around the mean and the minimum and maximum of the box plot. In fact, at T0 outliers emerged close to 0 and 100 (as minimum and maximum scores in NCS, respectively) in all competencies. However, in the ‘teaching–coaching’ dimension, the dispersion even at T3 was wide, with several outliers close to 0 and close to 100. Moreover, this competence appears to have a smaller variation from T0 to T3 than the others (Figure 1).

### 5.2 Qualitative Method: The Potential Role of Simulation

Nine potentialities have been identified regarding the role of simulation in supporting NGRNs during their transition to independent practice. According to the focus groups, simulation may support NGRNs in (1) deepening competencies in the nursing assessment; (2) dealing with emergency, specific, advanced and situational settings to address specific complexities; (3) knowing about and steps for handling frequent and infrequent situations; (4) deepening technical skills for which they do not feel adequately prepared; (5) reliving and reviewing complex situations in a controlled context (e.g., near errors and issues of the practice); (6) identifying priorities and making decisions to become an independent practitioner; (7) addressing complex relational and management issues; (8) developing teamwork skills; and (9) dealing with unpleasant situations that might be complex to handle in a real work context (Table S2).

However, simulation in the transition phase is effective when designed as (1) fully accessible to NGRNs, (2) from high (during the work initiation phase) to lower intensity (in the following months), (3) integrated within each unit and (4) as an element of a comprehensive strategy creating a supportive environment welcoming new graduate (Table S2). The first strategy focuses on ensuring the accessibility of simulations, providing NGRNs an opportunity that is usually reserved to students; secondly, the importance of offering simulations of varying intensity was emphasised, allowing participants to acquire skills gradually, with more intensive training in the first days after the work initiation and less intensive over the following months. Another key strategy was to integrate the simulation centre within the operational units, developing an educational alliance and allowing NGRNs to apply the skills learned in the real working environment or to discuss the situation lived in a protected environment. Finally, the potentialities of simulation should be implemented in a supportive environment for NGRNs, where preceptorship programmes are offered to foster their integration and professional growth within the organisation. These strategies were identified as crucial to ensure effective learning targeted at the specific challenges that NGRNs might face in their career path.

### 5.3 Data Integration

By integrating the quantitative and qualitative data, two competencies as described in NCS were not suggested as supported by the simulation, namely, ‘helping role’ and ‘teaching–coaching’ (Table 4); differently, in all remaining competence domains, the contribution of simulation resulted to be valuable. In the ‘therapeutic interventions’ of the NCS, new graduates have reported a lower perception of competencies in procedures, experiencing difficulties in the clinical practice especially after the first 15 days. Preceptors recognised the value of simulation in developing these competencies. Moreover, they underlined the potential role of simulation in addressing specific gaps also in the ‘working role’, ‘managing situations’, ‘diagnostic function’, ‘ensuring quality’ and in ‘diagnostic function’ dimensions of the NCS. Simulations could help boost confidence in practical skills, which new graduates identify as lacking in the first period; however, preceptors consider simulation to play an important role also in other transversal competencies. Allowing NGRNs to access sessions where the simulation centre has been integrated within the clinical units may also facilitate the development of soft skills.

**TABLE 4:** Data integration: Qualitative and quantitative findings joint display expressing the simulation potentialities in the transition phase according to the self‐assessed competences of newly graduates.

Simulation potentialities according to focus groups of preceptors	Nurse Competence Scale degree of competence in each domain as self‐assessed by NGRNs at T0, T1, T2 and T3				
Themes	Domains	Work initiation	After 15 days	After 3 months	After 5 months
Not emerged	Helping role	Sufficient	Sufficient	Good	Good
Not emerged	Teaching–coaching	Sufficient	Sufficient	Sufficient	Sufficient
1. Deepening assessment competencies	Diagnostic function	Sufficient	Sufficient	Sufficient	Good
2. Dealing with emergency, specific, advanced, and situational settings to address specific complexities of work contexts	Managing situations	Sufficient	Sufficient	Sufficient	Sufficient
3. Knowing about and steps for handling frequent and infrequent situations					
4. Deepening technical skills for which they do not feel adequately prepared	Therapeutic interventions	Insufficient	Insufficient	Sufficient	Sufficient
5. Reliving and reviewing complex situations in a controlled context	Ensuring quality	Sufficient	Sufficient	Sufficient	Sufficient
6. Supporting the competence to set priorities and undertake independent decisions	Working role	Sufficient	Sufficient	Sufficient	Sufficient
7. Addressing complex relational and management issues					
8. Developing teamwork skills					
9. Dealing with unpleasant situations					

Abbreviation: NGRNs, Newly Graduates Registered Nurses.

## 6 Discussion

To our best knowledge, this is the first study exploring the potential role of simulation in the transition phase from graduation into the first work experience by combining self‐assessment data collected longitudinally from NGRNs and qualitative data obtained from their preceptors. The increased challenges faced in the transition phase due to multifaceted issues—such as the limited time available to detect needs and provide on‐the‐job training due to the nurse shortage—call for innovative solutions in the field.

### 6.1 Self‐Assessed Competencies From the First Day of Work up to 5 Months

Findings provide insights into the progression of NGRNs' competencies, showing that their perceived levels evolve from the work initiation up to 5 months of work. The longitudinal data suggest a substantial decrease in the perceived competence within the first 15 days of work, which aligns with the concept of ‘reality shock’ (Kramer, Brewer, and Maguire 2013). This initial dip is followed by a progressive increase in competence levels, particularly after 3 months. Several studies (e.g., Lima et al. 2014; Wangensteen et al. 2012) have measured the level of competencies as perceived by NGRNs; however, only a few have provided data over time from the work initiation until the fifth month. When compared with these studies measuring the competencies of NGRNs longitudinally (Lima et al. 2016; Lindfors et al. 2022; Willman, Bjuresäter, and Nilsson 2020), our participants reported higher scores on average.

The ‘therapeutic intervention’, including actions to oversee patients' illness, condition or injury, has been reported as insufficient in the first months. The NGRNs perceived themselves to be not competent in such complex tasks, calling for support to master them properly. Moreover, in all NCS dimensions, NGRNs reported fewer competencies after 15 days of working, data that are not comparable with previous studies given that they performed the second measurement at 2–3 months (Lima et al. 2016; Lindfors et al. 2022; Willman, Bjuresäter, and Nilsson 2020). The decreased scores suggest that NGRNs, facing the work reality, reshape their self‐perceived competencies (Meretoja, Isoaho, and Leino‐Kilpi 2004); the decrease could reflect NGRNs difficulty in translating theoretical knowledge into clinical practice, especially in areas requiring immediate and decisive management, such as handling emergency situations or making clinical diagnoses. After 3 and 5 months, higher scores were reported, which may suggest progressive confidence in the expected competencies. Moreover, while an ample range of perceptions was reported from the first 15 days to 3 months, with several participants reporting competencies at the lowest and highest levels, a greater homogeneity emerged in the following months.

Overall, quantitative data suggest that, during the weeks of the transition, supplementing the preceptorship programme with individualised educational strategies especially in the first month is recommendable; furthermore, over time, the personalised approach seems to become less important given that participants tend to rank their competencies homogeneously.

### 6.2 The Potential Role of Simulation in the Working Transition According to Preceptors

Simulation may have several potentialities in supporting NGRNs during the working transition: some are close to technical skills, while others are closer to knowledge and attitudes, thus reflecting the entire set of competencies expected from NGRNs (Rabie, Rabie, and Dinkelmann 2020).

Nine different potentialities have emerged, only one aimed at developing knowledge (‘Knowing about and steps for handling frequent and infrequent situations’) while two focused on technical/clinical skills (‘Deepening assessment competencies’ and ‘Deepening technical skills’) and the remaining on transversal competencies (e.g., ‘Developing teamwork skills’). Overall, simulation may function as a supportive tool minimising the ‘reality shock’ experienced by NGRNs. Moreover, while NGRNs tend to focus on mastering technical skills (which are more visible and measurable) (Viottini et al. 2024), simulations have been suggested by preceptors to support a variety of competencies—mainly transversal such as handling complex situations, developing decision‐making abilities and interpersonal skills—especially in such cases where, at the unit level, the limited time may prevent any support or where the performances delivered by new graduates are perceived suboptimal by preceptors.

Specifically, simulations may help deepen assessment competencies that have been reported from sufficient to good in our sample. Assessing the needs of patients is fundamental to diagnose problems and make appropriate decisions: therefore, simulation may help NGRNs accelerate improvements in this competence with the aim to protect patients. Moreover, simulation has been suggested to support the development of ‘Managing situations’, ‘Ensuring quality’ and ‘Working role’ competencies, thus helping new graduates manage unpredictable and complex clinical situations, enhancing their ability to make rapid, effective decisions and to function as a nurse in the context. In the specific field of ‘Therapeutic interventions’, simulation was also suggested to deepen skills where NGRNs perceived themselves to be not prepared. This perception may be associated with the complexity of the interventions required by the specific unit, the limited exposure to such interventions during clinical rotations (Zito et al. 2024) or their uncertainty to perform skills alone, without the supervision ensured during the nursing education. However, the limited competence perceived should be supported early through simulations to minimise unnecessary stress among NGRNs and to prevent issues concerning patient safety.

Overall, preceptors see simulation not only as capable of addressing gaps in technical skills, but also of making NGRNs feel prepared and ready for situations requiring independent decision‐making and team dynamics management. Only in two competencies, ‘helping role’ and ‘teaching–coaching’, simulation was not suggested: the first was self‐assessed by NGRNs at the higher levels as compared to other dimensions, thus indicating that they may be prepared to master the expected role; the second, instead, may be not seen as a priority by preceptors, if considered as abilities achievable through experience and deepened knowledge of patients' educational needs.

To be effective, simulations should be accessible to NGRNs, with structure and unstructured opportunities, integrated within the unit to ensure that the specific needs of the context are addressed. Above all, simulations should be considered an element of a strategy aimed at welcoming and supporting NGRNs.

### 6.3 Strength and Limitations of the Work

The study has several limitations. Firstly, although all strategies recommended have been followed (e.g., preventing the failure to locate research participants, to contact research participants and to achieve cooperation), (de Leeuw 2005) a consistent drop‐out has emerged not investigated in the reasons (e.g., leaving the workplace) according to the confidentiality of the data collected. Moreover, the study may have been affected by the so‐called mass resignment characterising new graduates (Catarelli et al. 2023). Secondly, the sample size of the study was not established a priori, in line with the pragmatic approach of the investigation close to the real‐world. However, the limited sample and the attrition rates observed over the follow‐up period may limit the generalisability of the findings. Thirdly, the study has not considered the variability of the healthcare settings experienced by NGRNs in their transition, as well as lived by preceptors—which may differ in resources, staffing and patient clinical complexities. Furthermore, despite the use of a validated tool, the self‐assessed data collected with the NCS may have been biased by over‐ or underestimation of competencies; finally, we have considered as insufficient those competencies assessed with a score below 60 out of 100: this cut‐off may be considered restrictive as compared to the literature (Meretoja, Isoaho, and Leino‐Kilpi 2004).

## 7 Conclusions

Our study highlights the dynamic nature of NGRNs' competence development during the transition phase and the need for targeted educational interventions. Preceptors identified simulation as capable of promoting a broader, holistic approach to care rather than focusing exclusively on technical skills, suggesting a comprehensive role of simulation in the transition phase. Simulation may support preparedness in a range of competencies, such as performing diagnostic functions, managing situations, delivering therapeutic interventions, ensuring quality assurance and in the working role. However, no potentialities emerged in the ‘helping role’ and ‘teaching–coaching’ competencies.

To be effective, simulations should be accessible to new graduates, modulated from high to lower intensity; offered during the first months of their working experience, as integrated within the unit, in order to offer learning scenarios and experiences close to patients' profile, competence and scope of practice expected in each context. Moreover, simulation should be considered as part of a wider strategy planned for the NGRNs transition to the workplace. Future research is called to investigate the outcomes of simulation in the early phase of the transition and its effectiveness in accelerating the development of the expected competencies. Moreover, research should explore the long‐term benefits of simulation, for example, its role in reducing transition‐related stress and improving retention rates among NGRNs. By providing robust evidence on these aspects, future studies can help refine simulation programmes and ensure that they are optimally designed to support the early stages of NGRNs' professional development.

## Author Contributions

Conceptualization: A.P. and M.M.; data curation: M.M., S.D., L.G., I.A., A.B., D.C., A.I., I.M., E.M., S.M., T.P., E.V., S.C., and A.P.; formal analysis: S.D., L.G., S.C., and A.P.; methodology: S.D., L.G., S.C., and A.P.; project administration: M.M., S.D., L.G., I.A., A.B., D.C., A.I., I.M., E.M., S.M., T.P., E.V., S.C., and A.P.; software: L.G.; supervision: A.P., S.C., and M.M.; writing – original draft: M.M., S.D., L.G., I.A., A.B., D.C., A.I., I.M., E.M., S.M., T.P., E.V., S.C., and A.P.; writing – review and editing: M.M., S.D., L.G., I.A., A.B., D.C., A.I., I.M., E.M., S.M., T.P., E.V., S.C., and A.P.

## Conflicts of Interest

The authors declare no conflicts of interest.

## Statistics

The authors have checked to make sure that our submission conforms as applicable to the Journal's statistical guidelines. There is a statistician on the author team, Prof. Luca Grassetti.

## Supporting information


**Supplementary material**.

## Data Availability

The data that support the findings of this study are available from the corresponding author upon reasonable request.
