# Supplementary material for: The Role of the Simulation in Supporting Newly Graduated Nurses in Their First 5 Months of Working Transition: Findings From a Mixed‐Method Study
Source: J Clin Nurs. 2024 Dec 12;35(10):4210–20. doi: 10.1111/jocn.17617 (PMC13569388; doi:10.1111/jocn.17617)
Supplement: Supplementary file 1 [file JOCN-35-4210-s001.docx]

**Supplementary Table 1 -** Good Reporting of a Mixed Methods Study (O'Cathain, Murphy & Nicholl, 2008)

| **Criteria** | **Section where the data is reported** |
| --- | --- |
| (1) Describe the justification for using a mixed methods approach to the research question | Study Design |
| (2) Describe the design in terms of the purpose, priority and sequence of methods | Study Design |
| (3) Describe each method in terms of sampling, data collection and analysis | Settings and participants  Data Collection and procedures  Data Analysis |
| (4) Describe where integration has occurred, how it has occurred and who has participated in it | Integration of Data |
| (5) Describe any limitation of one method associated with the present of the other method | Limitations |
| (6) Describe any insights gained from mixing or integrating methods | Discussion |

O'Cathain, A., Murphy, E., & Nicholl, J. (2008). The quality of mixed methods studies in health services research. Journal of Health Services Research & Policy, 13(2), 92-98. https://doi.org/10.1258/jhsrp.2007.007074

**Supplementary Table 2 –** Trail Code of focus group narratives regarding the simulation potentialities during the transition phase

| **Themes** | **Examples of quotes** |
| --- | --- |
| 1. Deepening assessment competences | “Highlighting (in the assessment) situations that are instead to be managed, controlled, not to be missed”. (MA) |
| 2. Dealing with emergency, specific, advanced, and situational settings to address specific complexities of work contexts | “Knowing how to work ... when there is urgency. It’s important to know how to handle it with the people you have available”. (MA)  “... to train the new hires by simulating scenarios just with the life pack, the patient changing pace, or I don’t know and having the different types of patients. I don’t know the polytrauma rather than infarct rather than difficult intubation, etc.”. (ICU)  “to deal with emergency situations and for new hires …” (SA)  “However, also, how to say other situations that need instead of how to say, maybe a simulation, because anyway it has to be something a bit advanced”. (PA) |
| 3. Knowing about and steps for handling frequent and infrequent situations | “The not knowing well sometimes, because it’s not something that happens every day, the not knowing well what the things are, the steps that you have to do, also because then the anaesthesiologist comes to you”. (SA)  “Look, you’re going to get a situation where you have to study this, look at it now. I tell you what you’re going to be tested on that I would probably need to dilute an adrenaline ... I mean with the potential to then be able to have a more relaxed discussion”. (ICU)  “As a side activity to what is the direct experience in the operating room, regarding maybe sterility management rather than the various types of instrumentation that you may find in surgery, also in terms of what is then the speciality that you’re going to be instrumenting for”. (ICU) |
| 4. Deepening technical skills for which they do not feel adequately prepared | “If it is in need of trying, what do I know, placement of bladder catheter rather than peripheral venous catheter”. (MA)  “Vascular accesses, though even there, that is, we can’t have them tried on newborns”. (PA) |
| 5. Reliving and reviewing complex situations in a controlled context | “Because it means you don’t stop reality and relive it, you relive it though, in light of the reasoning that an experienced nurse does”. (MA) |
| 6. Supporting the competence to set priorities and undertake independent decisions | “… put the newly hired colleague in difficulty in the need to make decisions, to relate to someone they are in difficulty with ... So a hypothetical scenario that we can safely take from a department that may be the medical.” (MA)  “They look up to the experienced colleague; however, they don’t go into their thought process, no, they take what is obvious, thinking let’s say to create protected situations in which those who then follow you even in the ward, which may in fact help you understand what I’m seeing. What I’m thinking at this juncture, at a time that is as dedicated as the simulation, it certainly buys time, then the situation will happen.” (ICU)  “Also help them for, I don’t know, how to prioritize the activities to do for the patient”. (ICU) |
| 7. Addressing complex relational and management issues | “But the reality is to say, to manage a situation where the relational aspect also comes into play towards colleagues towards even how to say an actor, among whom could be the doctor, the colleague who does not speak to you, who does not greet you”. (MA) |
| 8. Developing teamwork skills | “Knowing how to work in the team no, when there is urgency. It is important to know how to manage it with the people you have available.” (MA) |
| 9. Dealing with unpleasant situations | “… maybe I would say on the new hires ... there is little awareness of the role that we have towards education precisely, but also intervention even in somewhat unpleasant situations, such as for example the patient presenting as very neglected, dirty, and smelly ...”. (SA) |
| **Strategies** | **Examples of quotes** |
| 1. Ensuring accessibility to simulation | “It is not accessible to professionals because they are not students”. (MA) |
| 2. Offering simulation from high (in the beginning phase) to less intensity (later) | “I would like to bring simulations, case simulations from A to Z, to the ER. In the meantime, we assess your basic background, a test, a whatever, then we do the mid-term training, what we decide to do, re-evaluate, see how we did.” (ICU)  “Proposed full-case simulations from start to finish to assess background, provide mid-term training, and evaluate learning”. (ICU)  “Just courses for the ward so that I can find out why I am doing those things there, because otherwise they just become mechanical actions”. (SA) |
| 3. Integrating the simulation centre with the units | “Thinking about situations that can be extremely contextualized, in the sense that the mannequin can be transported, and you can also transport it to a doctor’s room just to say no.” (MA)  “... I think it can in short then actually help to build something”. (ICU) |
| 4. Creating a supportive environment towards new graduates | “... but I’m telling you I’m struggling”. (SA) |

**Legend**: MA=medical area; SA=surgical area; PA=paediatric area; ICU=intensive care unit; NCS=Nursing Competence Scale; NGRNs, newly graduates register nurses.

**Supplementary Table 3**. Legitimation type, descriptions and strategies used (Onwuegbuzie, Johnson & Collins, 2011; Perez et al., 2023)

| **Legitimation Type** | **Description** | **How legitimation type was addressed** |
| --- | --- | --- |
| Sample integration | The degree to which appropriate conclusions are made from the quantitative and qualitative samples and appropriately integrated to produce quality meta-inferences. | The preceptors involved were appointed to supervise the NGRNs involved in the study during their working transition. |
| Emic-etic | The degree to which a researcher accurately depicts the insider’s (emic) and outsider’s (etic) perspectives in understanding or explaining a phenomenon. | The research team (see authors) were working at the hospital and in academic settings – as insiders and outsiders – thus providing an emic-etic perspective. |
| Weakness minimization | The degree to which weaknesses of one method are augmented by the strengths of another method in the study to realize the potential of MMR. | The quantitative data were collected from NGRNs, while an in-depth reflection was collected from focus groups, thus compensating for the potential lacks of the online survey, the completion of which required few minutes. |
| Multiple validities | The degree to which a researcher uses an appropriate *subset* or combination of the pertinent quantitative, qualitative, and mixed methods “validities” for a particular research study, to produce defensible/justified findings and high-quality meta-inferences. | The integration of the data, as reported in Table 4, provided valid findings. |
| Commensurability approximation | The degree to which the researcher makes cognitive switches from quantitative to qualitative lenses to yield syntheses and high-quality meta-inferences and a more nuanced worldview. | The research team was composed of multiple members – hospital- and academic-based – to provide a bilateral viewpoint, thus incorporating diverse perspectives from different members in the research team. |
| Conversion | The degree to which quantitizing and qualitizing data produced interpretable results and high meta-inference quality. | Table 4 provided transparent and interpretable results, derived from qualitative and quantitative data. |
| Paradigmatic/Philosophical | The degree to which a researcher successfully integrates their epistemological, ontological, axiological, methodological, and rhetorical beliefs underlying the quantitative and qualitative approaches into a workable mixed methods paradigm or research approach. | An independent analysis of the data was performed by different researchers (e.g. qualitative and statisticians) to address the value of each strand. |
| Multiple stakeholder | The degree to which the researcher has addressed the interests of different stakeholders (e.g., quantitative, qualitative, and MM researchers, policymakers, groups with little power) in the report and generation of high-quality meta-inferences. | Findings have been shared with the hospital community and the nurse educators of the nursing programs. |
| Sequential | The degree to which a later stage in a sequential design appropriately builds on an earlier stage, and researchers have addressed whether and how findings might have been different if the phases in the sequence were reversed. | The qualitative phase has been developed after the quantitative phase, the findings of which have been taken into consideration. |
| Integration[*](https://journals.sagepub.com/reader/content/189525f81df/10.1177/15586898221093872/format/epub/EPUB/xhtml/table3-15586898221093872.xhtml?hmac=1727258510-nCeUyCQelELrvKytrZ57cmPfJwYK5kXWtaIPiX2krO4%3D#table-fn2-15586898221093872) | The degree to which the researcher has successfully integrated quantitative and qualitative data, analysis, and conclusions. | A joint display was used to maximize integration and produce the findings. |
| Pragmatic[*](https://journals.sagepub.com/reader/content/189525f81df/10.1177/15586898221093872/format/epub/EPUB/xhtml/table3-15586898221093872.xhtml?hmac=1727258510-nCeUyCQelELrvKytrZ57cmPfJwYK5kXWtaIPiX2krO4%3D#table-fn2-15586898221093872) | The degree to which the purpose and goals of the research were met, the research problem was solved, the research questions were answered, and “actional results” were provided. | In the conclusions, the practical actions based upon the findings have been summarized. |

From: Onwuegbuzie, A. J., Johnson, R. B., & Collins, K. M. T. (2011). Assessing legitimation in mixed research: a new framework. *Quality and Quantity, 45*, 1253–1271. <https://doi.org/10.1007/s11135-009-9289-9>

Perez, A., Howell Smith, M. C., Babchuk, W. A., & Lynch-O’Brien, L. I. (2023). Advancing Quality Standards in Mixed Methods Research: Extending the Legitimation Typology. *Journal of Mixed Methods Research, 17*(1), 29-50. <https://doi.org/10.1177/15586898221093872>
